# Supplementary material for: Streamlining brain tumor surgery care during the COVID-19 pandemic: A case-control study
Source: PLoS One. 2021 Jul 29;16(7):e0254958. doi: 10.1371/journal.pone.0254958 (PMC8321144; doi:10.1371/journal.pone.0254958)
Supplement: Attachment — Submitted filename: Response to Reviewers PONE-D-21-14344 June 14 2021.docx [file pone.0254958.s002.docx]

June 14, 2021

Robert Jeenchen Chen, MD, MPH
Academic Editor
PLOS ONE

Re: Revision of manuscript PONE-D-21-14344: “**Streamlining brain tumor surgery care during the COVID-19 pandemic: a case-control study”**

Dear Dr. Chen:

Thank you for the helpful critique of our manuscript. Here in is our response to the reviewers’ critiques.

**Reviewer #1:** no response needed.

**Reviewer #2:**  The authors should include a more detailed description of post-operative management in order to reduce the need for ICU utilization. In fact, in MM section, they described a general protocol in which they stated that admittance to ICU is "generally reserved for patients with significant comorbidities, severe preoperative neurological deficits, severe preoperative brain edema, high seizure risk, new postoperative deficits, requiring continued mechanical ventilation or at significant risk of airway compromise". However, they did not specify if this somehow changed during pandemic period. Considering that case mix did not change in the pandemic period, I suppose that not only patient and family counseling was the key factor in reducing ICU admission. Was a dedicated protocol of recovery room monitoring of the patient applied (how many hours, what parameters, etc) in order to more accurately select patients really needing ICU? It has been already demonstrated in previous studies that this could reduce ICU utilization without impacting on post-operative complication and mortality.

**Response:** This is an excellent point – indeed part of the overall paradigm shift was enhanced observation in recovery room for the team to feel comfortable for patient recovery in a non-ICU bed. As such we have provided more detail on post-operative management as it relates to our protocol in the recovery room and on early patient assessment to determine if non-ICU admission was safe. These additions are in the abstract, author summary, methods (pages 8,10) and discussion (pages 18,20). We also modified Table 1 section 2, line a:“low likelihood of needing ICU observation” and added line b: “Recovery room assessment assuring safety of non-ICU admission.”

**Reviewer #3:** According to the description of the authors, this protocol was already used in the pre-pandemic epoch (Table 1). Yet, there were significant differences between these two cohorts in terms of rates of ICU utilization and length of hospital stay in this study. Therefore, these favorable trends cannot be attributed to the using of minimally invasive surgery, total intravenous anesthesia, less postoperative narcotics using and early postoperative imaging. Instead, more vigorous patient education and encourage of early mobilization were the key factors to make the differences. Therefore, discussion of this study should be focused on the elucidation of patient education enhancement and ways of promoting early ambulation.

**Response:** The reviewer is correct that our protocol of minimally invasive surgery, TIVA, complication avoidance protocols, early imaging, and limited narcotics use was already in place. We have already emphasized this point in the abstract conclusion, introduction, methods, discussion and Table 1. We stress that our reduced LOS and ICU use resulted from more extensive patient prep, team prep and awareness, more rapid patient mobilization and early contact with the patient and their caregivers on POD1 post-discharge. We clearly acknowledge that these practices were layered upon our existing foundational practice of minimally invasive surgery as detailed in Table 1 section 1. We also added as per Reviewer #2 that more rigorous recovery room assessment was utilized to assure safety of non-ICU admission.

**Reviewer #3:** The attached figure showing length of hospital stay of this study is ambiguous and cannot help the readers to understand this article better. The suggestion is either to delete this figure from this article or to redesign this bar chart with separated bars to illustrate the length of stay of these two patient groups underwent two different procedures. The unit of the length (day) also need to be specified.

**Response:** Regarding Figure 1, we have added the units (days) along the x axis (thank you for this suggestion). We appreciate the Reviewer’s concern and we have tried other iterations of this graph (including separating craniotomy and endonsal cohorts). However, we believe the current figure version adds an important and relatively clear visual representation of the data showing a favorable shift in LOS during the pandemic epoch. Given that Reviewers 1 and 2 did not have any issues with Figure 1, we request to leave it in the manuscript as is but modified with days added on x-axis.

We hope these revisions are acceptable.

Sincerely,

Daniel F. Kelly, M.D.
